# Mendelian randomization for cardiovascular diseases: principles and applications

**DOI:** 10.1093/eurheartj/ehad736

**Published:** 2023-12-14

**Authors:** Susanna C. Larsson, Adam S. Butterworth, Stephen Burgess

**Affiliations:** 1 Unit of Medical Epidemiology, Department of Surgical Sciences, Uppsala University, Uppsala, Sweden; 2 Unit of Cardiovascular and Nutritional Epidemiology, Institute of Environmental Medicine, Karolinska Institutet, Stockholm, Sweden; 3 British Heart Foundation Cardiovascular Epidemiology Unit, Department of Public Health and Primary Care, University of Cambridge, Cambridge, United Kingdom; 4 Victor Phillip Dahdaleh Heart and Lung Research Institute, University of Cambridge, Papworth Road, Cambridge, United Kingdom; 5 British Heart Foundation Centre of Research Excellence, School of Clinical Medicine, Addenbrooke’s Hospital, University of Cambridge, Cambridge, United Kingdom; 6 Health Data Research UK, Wellcome Genome Campus and University of Cambridge, Hinxton, United Kingdom; 7 NIHR Blood and Transplant Research Unit in Donor Health and Behaviour, University of Cambridge, Cambridge, United Kingdom; 8 MRC Biostatistics Unit, University of Cambridge, Cambridge, United Kingdom

**Keywords:** Cardiovascular disease, Genetics, Mendelian randomization, Single-nucleotide polymorphisms

## Abstract

Large-scale genome-wide association studies conducted over the last decade have uncovered numerous genetic variants associated with cardiometabolic traits and risk factors. These discoveries have enabled the Mendelian randomization (MR) design, which uses genetic variation as a natural experiment to improve causal inferences from observational data. By analogy with the random assignment of treatment in randomized controlled trials, the random segregation of genetic alleles when DNA is transmitted from parents to offspring at gamete formation is expected to reduce confounding in genetic associations. MR analyses make a set of assumptions that must hold for valid results. Provided that the assumptions are well justified for the genetic variants that are employed as instrumental variables, MR studies can inform on whether a putative risk factor likely has a causal effect on the disease or not. MR has been increasingly applied over recent years to predict the efficacy and safety of existing and novel drugs targeting cardiovascular risk factors, and to explore the repurposing potential of available drugs. This review article describes the principles of the MR design and some applications in cardiovascular epidemiology.

## Graphical abstract

**Graphical abstract:**
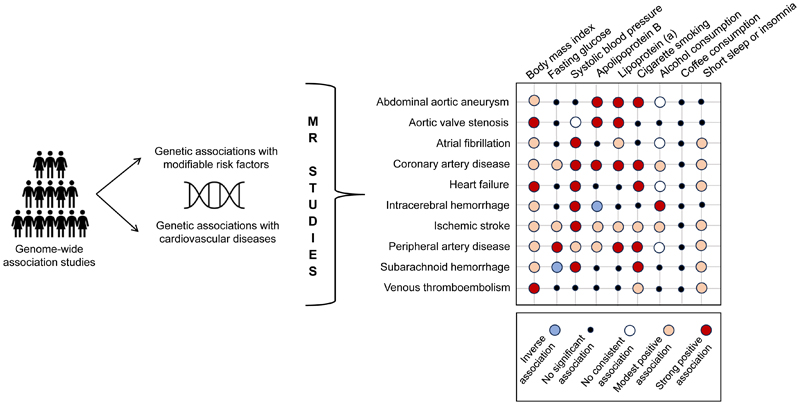
Mendelian randomization findings on major cardiometabolic and lifestyle factors and common cardiovascular diseases.

## Introduction

Identification of causal risk factors and effective treatments for prevention of cardiovascular disease (CVD), the leading cause of morbidity and premature death worldwide,^1^ is crucial from both individual and societal perspectives. Randomized controlled trials (RCTs) are considered the gold standard design to infer causality. However, RCTs are expensive, time consuming, and often unfeasible to conduct, for example because of poor long-term compliance and ethical issues about random treatment allocation. Thus, relationships of modifiable risk factors with CVD events have mostly been investigated using observational study designs, such as case-control and cohort studies, which cannot reliably infer causality as confounding and reverse causation bias can distort the findings.

Large-scale genome-wide association studies (GWAS) performed over the last decade have uncovered numerous genetic variants associated with cardiovascular risk factors, such as body mass index (BMI),^2^ glycemic traits,^3^ blood pressure,^4^ blood lipids,^5^ alcohol and tobacco use,^6^ coffee consumption,^7^ and physical activity,^8^ as well as CVD outcomes^9–14^ (Table 1). These discoveries have enabled the Mendelian randomization (MR) design, which employs genetic variation as a natural experiment to improve causal inferences from observational data.

This state-of-the-art review describes the principles and some applications of the MR design to improve causal inference in cardiovascular epidemiology. Information included in this review is based on literature published through 1 July 2023.

## What is a Mendelian randomization study?

**Figure 1:**
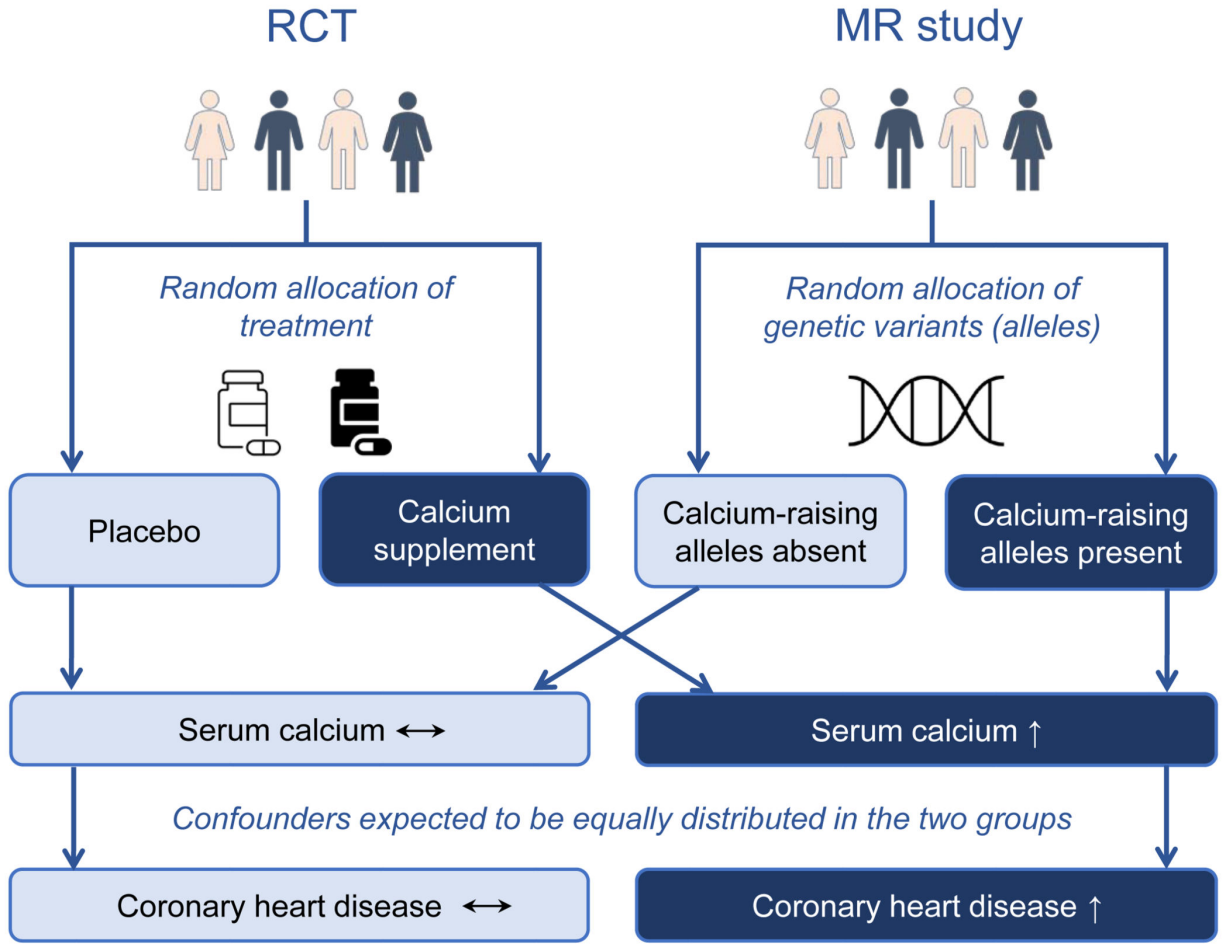
Comparison of randomized controlled trial (RCT) and Mendelian randomization (MR) study designs showing the common basis behind interpretation of a causal effect of higher serum calcium levels on coronary heart disease. According to Mendel’s laws, random and independent inheritance of genetic alleles can be thought of analogously to random allocation of treatment *vs*. placebo in RCT. Therefore, by the same reasoning, if MR finds genetic variants affecting serum calcium levels are associated with a difference in coronary heart disease risk, it provides evidence that serum calcium causally affects coronary heart disease.

MR is an application of instrumental variable analysis, which aims to test a causal hypothesis in non-experimental data. In an MR analysis, genetic variants, commonly single-nucleotide polymorphisms, are used as instrumental variables for the putative risk factor. The principle of MR refers to Mendel’s second law of independent segregation of genetic alleles when DNA is transmitted from parents to offspring at gamete formation. This is similar to the random assignment of treatment in an RCT, which aims to produce groups with similar clinical characteristics, hence reducing the risk of confounding. Figure 1 illustrates the analogy of an RCT and MR study investigating the effect of higher serum calcium levels on coronary heart disease risk. In this example, participants in the RCT are randomly assigned to receive either placebo or calcium supplements, leading to higher serum calcium levels in the treatment group.^15^ Analogously, in the MR study, the study population is ‘randomized’ by genetic variants that associate with serum calcium levels; for each variant, a participant may inherit the allele that raises serum calcium levels, or the allele that does not raise serum calcium levels. In both studies, the randomization is independent of confounding factors, and allows inferences on the effect of elevated serum calcium levels on coronary heart disease risk. If the randomized group in an RCT (or the genetically “randomized” group in MR) with higher average levels of serum calcium also have higher risk of coronary heart disease, this is indicative of a causal effect of calcium levels on coronary heart disease risk. An MR study also diminishes the risk of reverse causation bias as genetic variants are unchangeable and cannot be influenced by disease status. A glossary of common terms used in MR studies is provided in Box 1.

## What are the advantages?

MR studies have several advantages over RCTs. They are often faster and cheaper to conduct, as they can be conducted using existing large-scale GWAS data. MR studies can inform on potential causal relationships between modifiable risk factors and rare diseases that would require extensive sample sizes and long-term follow-up for sufficient endpoints to occur in an RCT. Moreover, MR studies can investigate exposures with expected adverse effects on disease risk, which would be unethical to test in trials. RCTs for a modifiable risk factor or medical treatment usually examine short-term effects as long-term compliance can be difficult to achieve and cost increases with longer duration. In contrast, as genetic variants are fixed at conception, MR results reflect the effects of life-long perturbations in the risk factor. Thus, MR is a valuable study design to overcome several of the limitations and problems confronted in conventional observational studies and RCTs. Nonetheless, MR should not be considered as a panacea as this design comes with its own set of assumptions and caveats, as described below.

## What are the assumptions?

**Figure 2:**
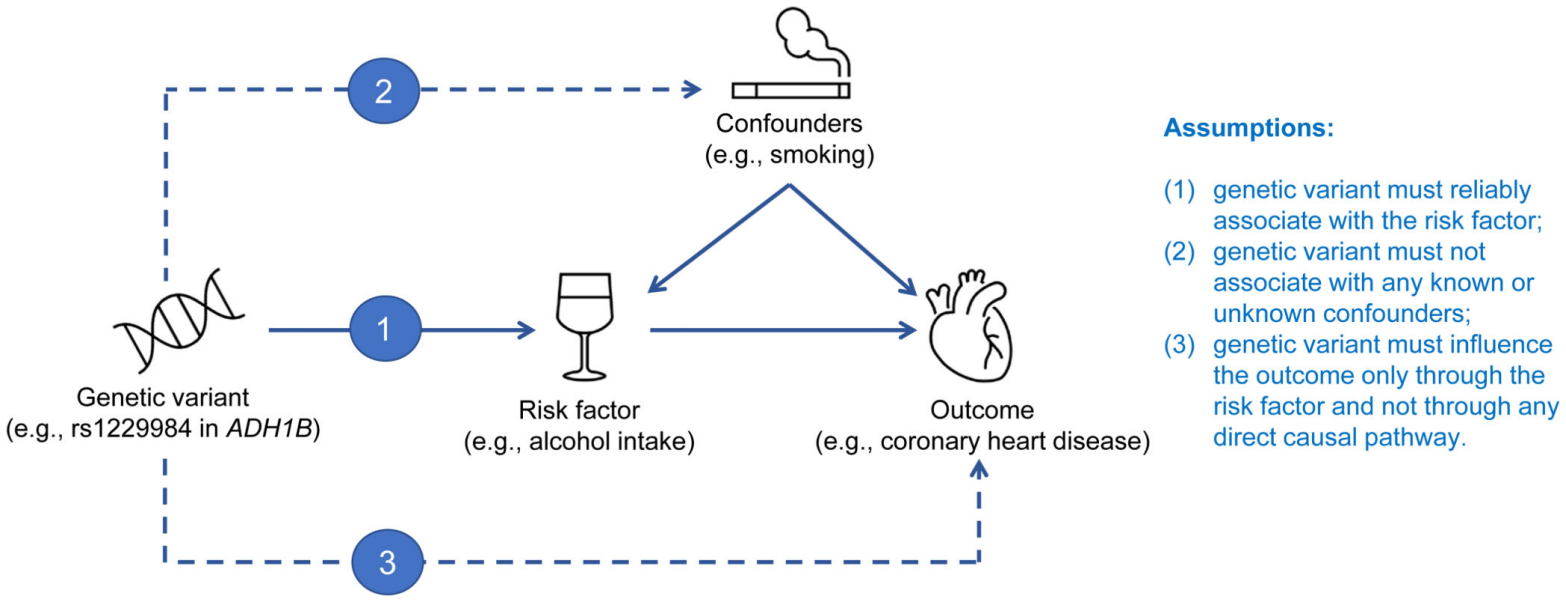
Illustration of the Mendelian randomization assumptions with the example of alcohol consumption as the putative risk factor and coronary heart disease as the outcome. Dashed lines indicate pathways that would violate the assumptions. In this example, a genetic variant in the alcohol dehydrogenase 1B (*ADH1B*) gene is robustly associated with alcohol consumption in individuals of European ancestries, is not associated with smoking (a main potential confounder) and has a key role in the metabolism of alcohol.

The three core assumptions that must hold for valid results in an MR analysis are illustrated in Figure 2. Specifically, the genetic variant (or multiple genetic variants) used as instrumental variable for the risk factor must: (1) reliably associate with the risk factor under investigation (relevance assumption); (2) not associate with any known or unknown confounding factors (independence assumption); and (3) influence the outcome only through the risk factor and not through any direct causal pathway (exclusion restriction assumption). The first assumption can be tested by choosing genetic variants that are significantly associated with the risk factor in a GWAS. Typically, genetic variants selected as instrumental variables are associated with the risk factor at the conventional level of genome-wide significance (*P*<5×10^-8^), although increasingly, Mendelian randomization analyses are conducted using variants from gene regions chosen based on prior knowledge about the relevance of the gene function to the risk factor. The plausibility of the second assumption can be evaluated by examining whether the genetic variant is associated with competing risk factors. The third assumption cannot be assessed directly but must be justified by biological knowledge. In MR analyses involving multiple genetic variants, the plausibility of the assumptions can also be assessed by statistical methods (e.g., MR-Egger test for pleiotropy, see Box 1).

## What are the caveats?

**Figure 3:**
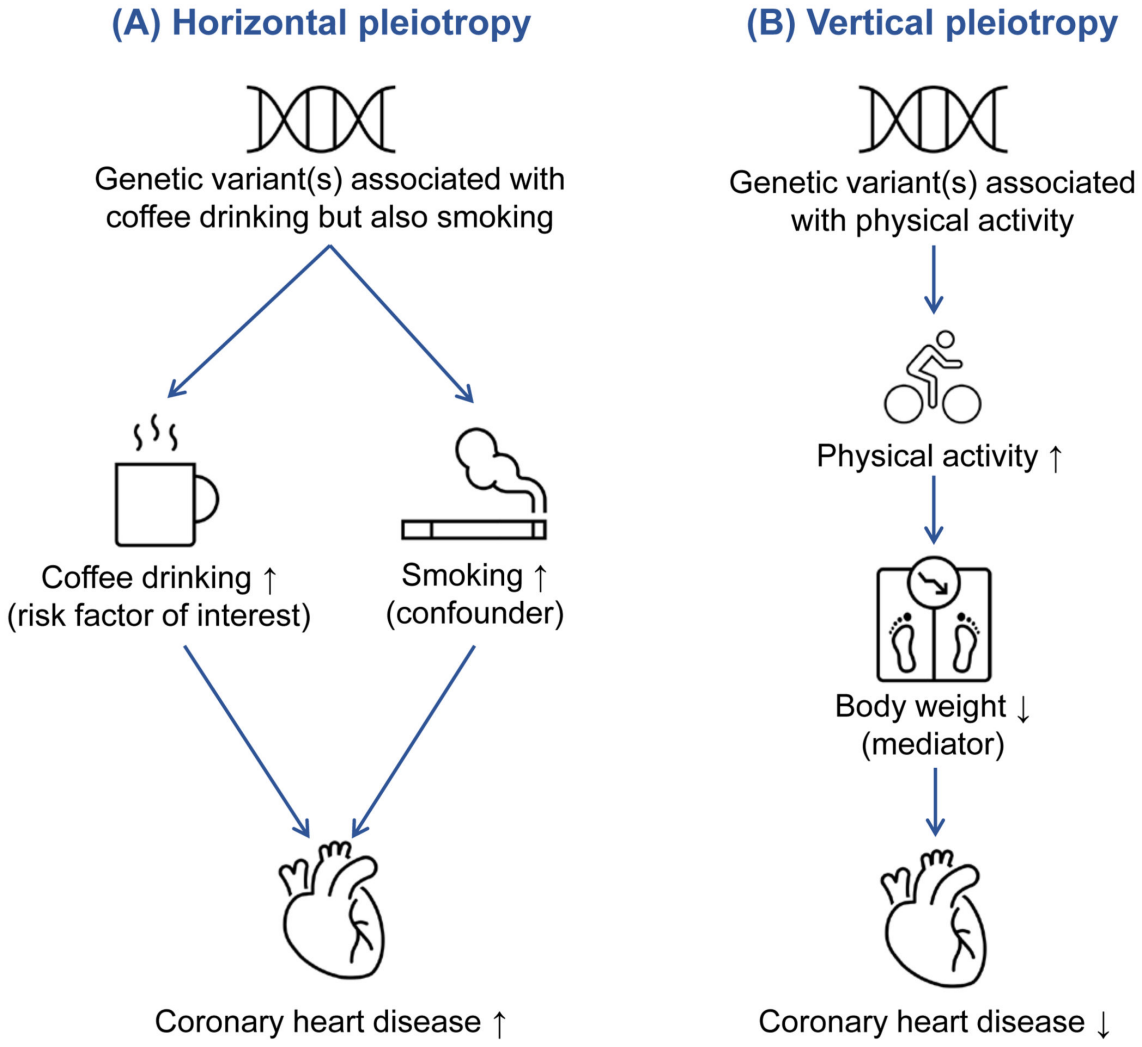
Examples of horizontal and vertical pleiotropy in a Mendelian randomization study. (A) An example of horizontal pleiotropy, in which the variant used as instrumental variable for coffee consumption is also associated with smoking (confounder), leading to violation of the third assumption (exclusion restriction) and can invalidate the results. (B) An example of vertical pleiotropy, in which the effect of physical activity on coronary heart disease is mediated by body weight. This does not distort the findings.

A primary concern to the validity of results from an MR analysis is pleiotropy, specifically ‘horizontal pleiotropy’ whereby a genetic variant affects the outcome through a pathway that does not involve the risk factor of interest. This would violate the MR assumptions and can be caused by multiple biological functions of the gene. It occurs where the variant associates with a factor (e.g., educational attainment) that is upstream of the risk factor of interest and which associates with multiple downstream risk factors (e.g., lifestyle factors) that affect the outcome via distinct pathways. Horizonal pleiotropy can produce a spurious, non-causal association between genetic predictors of the studied risk factor and the outcome but can also result in a false negative finding if the pleiotropic effect counteracts the true causal effect of the risk factor on the outcome. As an example, use of genetic variants that associate with coffee consumption (risk factor of interest) but also with another risk factor, such as smoking (confounder), that is not on the causal pathway from coffee consumption to coronary heart disease would give an estimate of the association between genetically predicted coffee consumption and coronary heart disease that does not correspond to the true causal effect (Figure 3).

Another type of pleiotropy, termed ‘vertical pleiotropy’ is when a genetic variant associates with another factor on the causal pathway from the genetic variants via risk factor to the outcome, such that any causal pathway from the variants to the outcome passes through the risk factor. This type of pleiotropy does not invalidate MR estimates. Indeed, some MR studies seek to uncover factors that lie on the causal pathway from the studied risk factor to disease, as these are potential mediators of the causal relationship and can improve our mechanistic understanding about causal pathways. As an example, the association between higher physical activity level and reduced risk of coronary heart disease may be mediated via BMI^8^ (Figure 3). However, distinguishing between horizontal and vertical pleiotropy is primarily dependent on our biological understanding of the relationships between the genetic variants, exposure, outcome, and pleiotropic factors.

Linkage disequilibrium (LD), which refers to the correlation of genetic variants in the population, is another potential caveat in MR studies. Genetic variants in physical proximity on the same chromosome can be in LD. Confounding would result if the genetic variant used to proxy the risk factor of interest is in LD (i.e., it is correlated) with another genetic variant that is associated with the outcome through a pathway that does not involve the risk factor of interest. As an example, a study of genetically predicted glucose-dependent insulinotropic polypeptide receptor (GIPR) agonism in relation to CVD occurrence showed that the association of higher GIPR-mediated fasting glucose-dependent insulinotropic polypeptide levels with coronary artery disease risk was not driven by *GIPR* variants but was the result of LD confounding between variants at the *GIPR* locus and a variant in *SNRPD2*, an established coronary artery disease risk locus.^16^

## What are the limitations?

A shortcoming of the MR design is that it can only be applied to risk factors for which suitable genetic variants are available. Genetic variants typically have a small effect on most risk factors (i.e., they explain a small proportion of the variation), which can lead to low statistical power in the MR analysis and the risk of false negative findings. The proportion of variance explained and thus the statistical power can be increased by utilizing multiple genetic variants associated with the risk factor as instrumental variables. For example, the fat mass and obesity-associated gene (*FTO*) is the locus with the largest effect on BMI, but this locus explains less than 0.5% of the variation in BMI in populations of European ancestries and even less in populations of other ancestries.^17^ The corresponding variation explained by all near-independent genetic variants (n=941) found to be associated with BMI in a GWAS meta-analysis involving ~700 000 European ancestry individuals was ~6%.^2^ The amount of variation explained by known genetic variants is often below 5% for complex phenotypes. MR studies of such phenotypes require very large sample sizes, particularly large numbers of cases, to achieve reasonable power to detect weak to modest effects. The variation in the risk factor explained by genetics is higher for risk factors that are less influenced by environmental factors. As an example, circulating lipoprotein(a) (Lp[a]) levels are mainly determined by genetic variations at the *LPA* locus. Genetic variants that have been used to proxy the effect of Lp(a) explain over 60% of the variation in Lp(a) levels.^18^

## One- or two-sample MR study?

In a one-sample MR study, the genetic variant-risk factor association and the genetic variant-outcome association are obtained from the same individuals, while in a two-sample MR study those associations come from independent study populations. For example, a two-sample MR study on serum calcium levels and coronary heart disease risk can involve summarized (i.e., aggregated) data for the genetic associations with serum calcium from one study^19^ and the corresponding data for the genetic associations with coronary artery disease from another study.^20,21^ An advantage of the two-sample design is that statistical power is typically greater as existing summarized data from large-scale GWAS consortia can be used. The two-sample design comes with the requirement that the two samples represent similar underlying populations (or better still, the same population) as the genetic variants identified to the associated with the risk factor in the first sample should be reliable predictors of the risk factor also in the outcome dataset. This assumption may not hold if age, sex, ancestry, or other characteristics differ in the two samples. For example, genetic variants associated with smoking heaviness in a GWAS analysis involving smokers only would be unsuitable as instrumental variables in an MR analysis with outcome data from a population largely consisting of non-smokers. Ideally, there should be no overlap of the populations in the two samples as overlap of cases can bias MR estimates in the direction of the observational association, especially when the genetic associations with the risk factor are not strong.^22^ A limitation of using summarized data is the reliance on the validity of GWAS results reported by other research groups, and reduced flexibility in the MR analysis. Using data at the individual-level enables more comprehensive analyses, such as non-linear MR analysis or analysis of a specific subgroup (e.g., among smokers only). GWAS data that can be used in two-sample MR analyses are publicly available for many phenotypes and disease outcomes. A few examples of large-scale GWAS studies are listed in Table 1.

## How to select genetic variants?

There are two typical strategies for selection of genetic variants for use in a MR analysis. Genetic variant selection can be based on biological rationale or by including all independent genetic variants associated with the risk factor irrespective of biological function. For example, MR studies of the association between alcohol consumption and CVD have either used variants in genes that encode enzymes with a key role in alcohol metabolism,^23,24^ or all independent genetic variants associated with alcohol consumption at the genome-wide significance level in large consortium data.^6^ Alcohol (ethanol) is metabolized in the liver via two steps: firstly by alcohol dehydrogenases and secondly by acetaldehyde dehydrogenases. Genetic variants in the coding gene regions of these enzymes affect alcohol drinking behaviors as accumulation of the intermediate product of this two-step reaction (i.e., acetaldehyde) produces discomforts, such as facial flushing and increases of pulse rate and skin temperature, at sufficient concentrations.^25^ MR studies have demonstrated that higher alcohol consumption proxied by one or more variants in the coding gene regions for the alcohol or acetaldehyde dehydrogenases is associated with higher systolic blood pressure (SBP)^23,24,26,27^ and increased risk of coronary heart disease^23,27^ and stroke.^24,27^

Alcohol drinking behavior and many other phenotypes are polygenic, meaning that they are influenced by variants in many genes. When multiple genetic variants are available as instruments, with individual-level data, the variants can be combined into a polygenic score, which is the weighted sum of genotypes over many variants.^28^ This score can then be used as an instrumental variable. In a two-sample MR analysis based on summarized data, each variant provides its own MR ratio estimate that are combined by taking a weighted average of them. Similar to MR analyses of alcohol consumption proxied by variants in genes involved in alcohol metabolism, a two-sample MR analysis showed that higher alcohol consumption proxied by 94 genetic variants was associated with higher SBP and stroke risk.^27^

The two strategies to select genetic variants come with different strengths and limitations. The advantage of using few genetic variants with a clear biological role in influencing the putative risk factor is that the likelihood of pleiotropic effects is typically lower. For example, the instrument comprising all genetic variants associated with alcohol consumption was associated with smoking liability in UK Biobank.^27^

An advantage of using all genetic variants associated with the risk factor is that statistical power can be greater as the proportion of variation in the risk factor increases with the number of genetic variants employed as instrumental variables. Furthermore, when many genetic variants are available, a broad range of sensitivity analyses can be employed to test the MR assumptions.

## How to analyze the data and obtain causal estimates?

For a single genetic variant, the MR estimate can be obtained by dividing the variant-outcome association by the variant-risk factor association. The ratio is known as the Wald estimate. In a two-sample MR study based on multiple genetic variants and summarized data, the causal estimate can be obtained by the inverse-variance weighted method, which is a meta-analysis of the single Wald ratios and is the most efficient method (greatest statistical power) but is sensitive to pleiotropy.^29^ Several other methods that are more robust to pleiotropy but typically less efficient, such as the weighted median,^30^ MR-Egger,^31^ and MR-PRESSO^32^ methods, are commonly used as sensitivity analyses. These approaches require the availability of variants in multiple gene regions. A brief description of some commonly used MR methods is available in Box 1; further detailed comparisons of methods can be found elsewhere.^33,34^

## Why account for other risk factors?

According to Mendel’s laws, each characteristic should be inherited independently of other characteristics, thereby preventing confounding. Nevertheless, genetic variants may still have pleiotropic associations with other variables. Adjustment for related traits with shared genetic predictors and for known pleiotropic factors can be done in a multivariable MR analysis, which is a statistical approach that allows for the association of genetic variants with multiple risk factors to be incorporated into the analysis.^35^ As an example, multivariable MR analysis has been conducted to unravel which one (or more) of the atherogenic lipid-related traits accounts for the causal association with major CVDs. These studies have demonstrated that low-density lipoprotein cholesterol (LDL-C), apolipoprotein B, and triglycerides were all associated with coronary artery disease and ischemic stroke when assessed individually in univariable MR analysis.^36,37^ Nevertheless, only apolipoprotein B remained robustly associated with these CVDs in multivariable MR analysis with mutual adjustment for the other lipid-related traits.^36,37^

Multivariable MR analysis can also be applied to explore mediating effects of factors that may lie in the causal pathway from the studied risk factor to the outcome. As an example, multivariable MR analysis was performed to evaluate the mediating effects of cardiometabolic risk factors on the association between adiposity and common atherosclerotic CVDs.^38^ The study showed that genetically predicted SBP and type 2 diabetes liability mediated 27% and 41%, respectively, of the association between genetically predicted BMI and risk of coronary artery disease.^38^ Adjustment for blood lipids and smoking liability through multivariable MR analysis resulted in only minor attenuations in the association estimate for genetically predicted BMI in relation to coronary artery disease,^38^ suggesting that lipids and smoking were not major mediators or confounders of the relationship.

## Triangulating the evidence

Although MR studies can add an important piece to the puzzle on the possible causal effect of a risk factor on a health outcome, MR findings should be interpreted in the light of evidence from other sources such as traditional observational and experimental studies. As an example, results from prospective cohort studies have shown that high circulating calcium levels are associated with an increased risk of myocardial infarction.^39,40^ Furthermore, a meta-analysis of three RCTs showed that relatively short-term high-dose calcium monotherapy or calcium plus vitamin D supplements, both of which result in a slight but significant increase in serum calcium levels^15^ and are amongst the most commonly prescribed therapeutics,^41^ increased the risk of myocardial infarction.^42^ On top of this evidence, MR investigations have found that genetically predicted lifelong higher serum calcium levels are associated with an increased risk of coronary artery disease,^21^ myocardial infarction,^21^ and overall CVD.^42^ Hence, triangulating the evidence across study designs supports a causal association between short-term and lifelong modest elevations in circulating calcium levels and a higher risk of myocardial infarction.

## What are the applications?

MR has been applied to investigate potential causal relationships between putative risk factors and CVD risk as well as to predict the efficacy and adverse effects of existing and novel drugs and for drug repurposing opportunities. A summary of MR studies on conventional cardiovascular risk factors and lifestyle factors in relation to CVD risk is presented below and in the *Graphical Abstract*. Some examples of drug-target and drug repurposing MR studies are also provided.

## Conventional cardiovascular risk factors

MR studies have provided convincing evidence that greater adiposity, instrumented by BMI-associated genetic variants discovered by the Genetic Investigation of Anthropometric Traits consortium,^2,43^ is causally associated with increased risk of most CVDs.^13,44–48^ In a recent meta-analysis of MR studies, genetically predicted higher BMI was associated with an increased risk of all 14 studied CVDs.^46^ Likewise, genetically predicted greater waist-to-hip ratio, whole-body fat mass, and visceral fat, are associated with increased risk of CVDs.^38,49–52^ Genetically predicted adiposity is associated with cardiometabolic factors, including glycemic traits, blood pressure, and circulating lipids.^53–56^ MR studies have provided evidence that higher fasting insulin, fasting glucose, or glycated hemoglobin levels are causally associated with an increased risk of some CVDs (e.g., coronary artery disease, peripheral artery disease, and ischemic stroke),^48,57–59^ and that elevated SBP is a risk factor for most CVDs.^45,48,60^ With respect to circulating lipids, MR studies have concluded that atherogenic lipid-related entities, including LDL-C,^13,61–64^ apolipoprotein B,^13,36,37,61,65,66^ and Lp(a),^13,18,61,67–72^ are associated with an increased risk of atherosclerotic CVDs and that genetically predicted Lp(a) levels are associated with atrial fibrillation risk.^72^ Multivariable MR analyses have suggested that the associations of LDL-C with risk of coronary artery disease, ischemic stroke, and peripheral artery disease are largely driven by apolipoprotein B.^36,37,65^ MR findings have not supported an independent causal role of high-density lipoprotein cholesterol in major CVDs after accounting for LDL-C or apolipoprotein B.^36,37,48,61,65^

## Lifestyle factors

The MR design has been used to investigate the potential causal associations of lifestyle factors, including smoking, alcohol and coffee consumption, physical activity, and sleep patterns, with risk of CVD. A consistent association has been reported for genetic liability to smoking with increased risk of most CVDs,^48,59,73–75^ with the strongest magnitude of association observed for peripheral artery disease and abdominal aortic aneurysm.^73–75^

In contrast to conventional observational studies showing a protective association between moderate alcohol consumption and risk of coronary heart disease^76^ and ischemic stroke,^77^ MR studies have shown that genetically predicted higher alcohol consumption is associated with an increased risk of coronary heart disease^23,78^ and stroke^27,78^ in European populations. Moreover, in the China Kadoorie Biobank, alcohol consumption proxied by a loss-of-function variant of the aldehyde dehydrogenase 2 gene (common in east Asian populations) and a variant of the alcohol dehydrogenase 1B gene had a continuous positive log-linear association with risk of both ischemic stroke and intracerebral hemorrhage but was not associated with myocardial infarction.^24^ A nonlinear MR analysis in the UK Biobank showed that light alcohol drinking was associated with a minimal increase in coronary artery disease risk and that the risk increased exponentially at higher intakes.^78^ MR studies have provided suggestive evidence that genetically predicted higher alcohol consumption is associated with increased risk of abdominal aortic aneurysm,^27^ atrial fibrillation,^27,78^ heart failure,^78^ and peripheral artery disease,^27,59^ but not with aortic valve stenosis and venous thromboembolism^27^ in European populations.

The observational findings of an inverse association between moderate coffee consumption and risk of CVD,^79,80^ particularly coronary heart disease and ischemic stroke,^79^ have no support from MR studies which have proxied coffee consumption by a couple of variants in genes known to be involved in caffeine metabolism (and associated with coffee consumption)^48,80^ or all variants strongly associated with coffee consumption.^48,81^ Furthermore, no association has been observed between genetically predicted plasma caffeine levels and CVD risk.^82^ It should be noted that MR analyses have assumed a linear relationship between coffee consumption and CVD and results might therefore have been attenuated to the null if the relationship is nonlinear as suggested by observational studies (lowest CVD risk at 3 to 5 cups per day).^79^ The disparate results, which highlight the importance of triangulation of evidence, might also reflect residual confounding in the observational studies or pleiotropy in MR studies.

Observational findings of inverse associations between physical activity and risk of major CVDs^83^ have gained little support from MR studies. Suggestive evidence of strong inverse associations has been observed for genetically predicted vigorous physical activity and risk of myocardial infarction^84^ and for moderate-to-vigorous physical activity and risk of subarachnoid hemorrhage.^48^ However, other MR studies reported no association of genetically predicted self-reported moderate to vigorous physical activity with risk of coronary artery disease,^85^ ischemic stroke,^85^ peripheral artery disease,^59^ or heart failure.^86^ Likewise, no association has been found between genetically predicted accelerometer-based physical activity and risk of coronary artery disease or ischemic stroke.^85^ The genetic instruments used in these MR studies explain little variation in the physical activity phenotypes (i.e., between ~0.1% to 0.24%).^59,85,86^ Thus, the negative MR findings may reflect insufficient power to detect weak to modest associations.

The associations of sleep traits, particularly sleep duration and insomnia, with risk of CVD have been investigated in several MR studies.^48,59,87–90^ For example, an MR study involving 404 044 UK Biobank participants found that genetic liability to short sleep duration (≤6 h) was associated with increased risk of hypertension, coronary artery disease, myocardial infarction, and pulmonary embolism, and possibly with atrial fibrillation.^87^ Other MR studies found that genetic liability to short sleep duration was associated with an increased risk of peripheral artery disease.^59,88^ Moreover, genetic liability to insomnia has been found to associate with increased risk of several CVDs, including coronary artery disease, peripheral artery disease, atrial fibrillation, heart failure, ischemic stroke, and subarachnoid hemorrhage.^48,59,89,90^

## Predicting efficacy and adverse drug effects

The methodology of drug-target MR analysis to evaluate efficacy and safety of drugs targeting cardiovascular risk factors has been described in depth previously.^91–93^ In brief, most drugs act by targeting proteins, which are coded for by genes. Variants in the region of the relevant protein-coding gene can thus be used to proxy the pharmacologic effects of perturbing the corresponding drug target. Whereas MR studies of modifiable risk factors generally employ variants from multiple gene regions, MR analyses exploring drug target effects typically utilize variants from a single gene region, specifically, variants within the region around the protein-coding gene. Such an analysis is known as *cis*-MR analysis as variants near the protein-coding gene are named *cis*-variants. A prerequisite is that the selected genetic variants represent the clinical effects of the drug target.

As an example, MR has been applied to predict the effects of cholesterol-lowering drugs that target 3-hydroxy-3-methylglutaryl-CoA reductase (HMGCR; target of statins), Niemann-Pick C1–like 1 (NPC1L1, target of ezetimibe), proprotein convertase subtilisin/kexin type 9 (PCSK9, target of PCSK9 inhibitors), and cholesteryl ester transfer protein (CETP, target of CETP inhibitors) on CVD risk.^62,64,66,94–100^ In one of these initial MR studies,^94^ the researchers selected variants within ±100 kb of the *HMGCR* and *NPC1L1* genes that associated with LDL-C levels at a threshold of *P*<5.0×10^-6^ in the Global Lipids Genetic Consortium^101^ as instrumental variables to predict the corresponding drug effects. The analysis showed that genetically predicted LDL-C lowering mediated by variants in the *HMGCR* or *NPC1L1* gene or in both genes was associated with a reduced risk of coronary heart disease.^94^ Similar associations have been reported for genetic mimicry of HMGCR inhibition in relation to risk of ischemic stroke^96^ and abdominal aortic aneurysm.^64^ Likewise, genetic mimicry of PCSK9 or CETP inhibition has been found to associate with a reduced risk of coronary artery disease^62,98,99^ and abdominal aortic aneurysm^64^ as well as ischemic stroke in some^98,99^ but not all studies.^62,97,100^ With respect to adverse effects, genetically predicted LDL-C lowering mediated through variants in the *NPC1L1* gene has been associated with an increased risk of gallstone disease,^102^ whereas genetic mimicry of HMGCR and CETP inhibition has been associated with an increased risk of intracerebral hemorrhage^66^ and age-related macular degeneration,^100,103^ respectively. Additionally, genetically predicted LDL-C lowering independent of drug target has been reported to associate with an increased risk of type 2 diabetes.^95,104,105^

As a further example, *cis*-MR analyses have been performed to predict the cardiovascular effects of lowering circulating levels of Lp(a). These MR studies have used variants in the *LPA* gene region as instrumental variables and consistently demonstrated that genetically predicted higher Lp(a) levels are associated with an increased risk of many CVDs, particularly coronary artery disease, peripheral artery disease, aortic stenosis, abdominal aortic aneurysm, and ischemic stroke.^18,67–71^ According to a multivariable MR analysis, the increased risk of coronary artery disease related to higher Lp(a) levels is independent of apolipoprotein B.^106^ A recent study that integrated MR and phenome-wide association study technologies (MR-PheWAS; see Box 1) to explore the effects of Lp(a) on 1081 outcomes amongst ~400 000 participants of the UK Biobank found little evidence of adverse effects of lowering Lp(a) levels.^71^ Nevertheless, earlier MR studies have suggested a possible increased risk of Alzheimer’s disease associated with Lp(a)-lowering.^68,70^ RCTs are underway to investigate whether Lp(a)-lowering therapies can reduce the risk of recurrent major adverse cardiovascular events as well as to evaluate the safety and tolerance of such therapies.^107^

An important limitation of this approach is that the extent to which genetic variants mimic the action of specific drugs is often unclear.^108^ Further limitations are that genetic variants have life-long effects, whereas trials assess the impact of short-term interventions; trials often compare the impact of interventions on top of standard care (such as statins), and hence MR investigations may not reflect real-world practice; and trial endpoints often differ from outcomes in MR analyses.

## Drug repurposing

MR has been applied to evaluate the repurposing potential of available drugs. As an example, MR studies have demonstrated that genetic mimicry of interleukin-6 receptor blockade (targeted by tocilizumab) is associated with lower risk of rheumatoid arthritis,^109,110^ but also with reduced risks of several CVDs^110–115^ as well as COVID-19.^116^ Nonetheless, a possible side effect is an increased risk of pneumonia.^110,116^ These findings suggest that blockade of the interleukin-6 signaling pathway may be a target for the prevention of diverse CVDs and COVID-19 but that caution should be taken with regard to possible adverse effects.

As another example, the broad effects of genetic mimicry of tyrosine kinase 2 (TYK2) inhibition (targeted by deucravacitinib) on ~1500 outcomes amongst ~340 000 participants of the UK Biobank study was recently examined in an MR-PheWAS.^117^ The study showed that TYK2 inhibition instrumented by a variant in the *TYK2* gene was, as expected, effective in reducing the risk of psoriasis and other autoimmune diseases, but was associated with potential adverse effects such as increased risk of prostate and breast cancer.^117^

## Future directions

MR investigations are dependent on the availability of studies with linked genetic and epidemiological data. These have expanded in several directions in recent years: in size, in coverage, and in scope. Larger sample sizes enable more powerful analyses, as well as adequately powered analyses in population subgroups. Data are becoming available on a wider range of population groups, such as the multi-ancestry GWAS from the Global Lipids Genetic Consortium.^5^ This is important not only to improve representation in research findings, but also because key treatment-mimicking variants may only be available in specific ancestry groups, such as loss-of-function variants proxying darapladib in East Asians.^118,119^ Most GWAS and MR analyses conducted to date have included participants of primarily European ancestries. The generalizability of the findings to other ancestries deserves further study. Finally, ever more detailed data on proteomics,^120^ metabolomics,^121^ transcriptomics,^122^ and other biological domains are enabling focused, translational analyses to understand the potential effects of diverse interventions. This is combined with methodological innovations, enabling analyses that characterize causal non-linear response curves,^123^ identify relevant causal traits,^124^ and model effects on multiple outcomes.^125^

## Conclusions

MR analyses can provide critical evidence on the potential causal effects of many modifiable exposures, including traditional epidemiological risk factors, lifestyle factors, and druggable targets. The validity of inferences is subject to untestable assumptions that will not hold in all cases. Still, MR can add important evidence supporting or dampening enthusiasm for the exposure as a worthwhile target for therapeutic intervention.

## Figures and Tables

**Table 1:** Examples of genome-wide association studies relevant to cardiovascular research

Phenotype	Consortium or study	No. of genetic variants or loci*	Total sample size (cases)
***Potential risk factors***			
Alcohol and tobacco use	GSCAN	378/99^†^	Up to 1 232 091^6^
Blood lipids	GLGC	773 loci	Up to 1 654 960^5^
Blood pressure traits	ICBP and UKBB	>1000 variants	757 601^4^
Body mass index	GIANT and UKBB	941 variants	681 275^2^
Coffee consumption	CCGC	8 loci	91 462^7^
Glycemic traits	MAGIC	242 loci	281 416^3^
Physical activity	51 studies	11 loci for MVPA	Up to 703 901^8^
***Cardiovascular outcomes***			
Aortic valve stenosis	Ten studies	18 variants	653 867 (13 765)^13^
Atrial fibrillation	AFGen and five other studies	142 variants	1 030 836 (60 620)^10^
Coronary artery disease	CARDIoGRAMplusC4D	241 loci	1 165 690 (181 522)^11^
Heart failure	HERMES and MVP	39 variants	1 188 957 (90 653)^14^
Stroke	GIGASTROKE	89 loci	1 614 080 (110 182)^12^

ICBP, International Consortium of Blood Pressure Genome Wide Association Studies; CARDIoGRAMplusC4D, Coronary ARtery DIsease Genome wide Replication and Meta-analysis plus The Coronary Artery Disease Genetics consortium; CCGC, Coffee and Caffeine Genetics Consortium; GIANT, Genetic Investigation of ANthropometric Traits; GLGC, Global Lipids Genetics Consortium; GSCAN, GWAS and Sequencing Consortium of Alcohol and Nicotine use; MAGIC, Meta-Analysis of Glucose and Insulin-related Traits Consortium. MVP, Million Veteran Program; MVPA, moderate-to-vigorous intensity physical activity; UKBB, UK Biobank. ^*^Number of independent or near-independent genetic variants (single-nucleotide polymorphisms) or loci identified to be associated with the phenotype at the genome-wide significance threshold. †Number of near-independent genetic variants associated with smoking initiation/alcohol consumption.

## Data Availability

All data in this paper is available via published articles.

## Box 1 Glossary of frequently used terms in Mendelian randomization studies

**Concepts**

- *Causality* − Refers to a cause-and-effect relationship: altering the level of the exposure would change the outcome (or change the risk of the outcome where it is a disease). In contrast, an ‘association’ does not necessarily imply causality but merely that the exposure and outcome are correlated.
- *Confounding* − Refers to a distortion in the estimate for a risk factor-outcome association that occurs when the risk factor of interest is associated with another factor that causally affects the outcome. For example, the association of alcohol consumption with coronary heart disease risk may be confounded by the fact that people who drink alcohol are also more likely to smoke cigarettes, which has a causal influence on disease risk.
- *Genome-wide association study* (GWAS) − A hypothesis-free study design that tests the associations of thousands or millions of genetic variants with a phenotype. The principal aim of a GWAS is to identify variants that are associated with the phenotype, which can be used to identify genes that are relevant to the etiology of the phenotype or to develop a predictive polygenic score for the phenotype.
- *Linkage disequilibrium* (LD) − Refers to the non-independent segregation of genetic variants. Genetic variants in proximity on the same chromosome can be inherited together, which can lead to correlations between them if allele frequencies are similar.
- *Mendelian randomization* (MR) − The use of genetic variants associated with the exposure (proposed risk factor) to understand the causal effect of the exposure on a health outcome.
- *Mendelian randomization phenome-wide association study* (MR-PheWAS) − A hypothesis-free study design that performs Mendelian randomization for a risk factor on a wide range of outcomes. A limitation of this approach is multiple hypothesis testing, which leads to a challenge in identifying true associations and biologically relevant associations.
- *Phenotype* − Refers to an individual’s observable characteristics, such as eye color, blood type, and body weight. An individual’s phenotype may be determined by genotype alone (e.g., in the case of blood type) or by both genotype and environmental factors (e.g., for body weight).
- *Pleiotropy* − Refers to the association of a genetic variant with multiple phenotypes. Horizontal pleiotropy refers to the association of a genetic variant with more than one phenotype on discrete biological pathways. This pleiotropy is of concern as it violates the exclusion-restriction assumption and can distort the results. Vertical pleiotropy refers to the association of a genetic variant with more than one phenotype on the same biological pathway, which does not invalidate the findings.
- *Reverse causation (also known as reverse causality) –* A phenomenon by which the outcome (disease) affects the levels of the exposure (risk factor) rather than vice versa, as would be expected. This bias is minimized in MR studies as genetic variants are unchangeable and cannot be influenced by disease status.
- *Single-nucleotide polymorphism* − A common genetic variation in which one base in the DNA is changed (e.g., a C instead of a T at a particular place in the genetic sequence).

**Statistical methods**

- *Inverse-variance weighted method* − Most efficient (greatest statistical power) and usually the main analytical method in MR studies involving multiple genetic variants. Requires that all genetic variants are valid instrumental variables.
- *Weighted median* − A common complementary method in MR studies that operates by taking the median of variant-specific estimates. Robust to outliers but sensitive to the addition or removal of genetic variants.
- *Multivariable MR* − A statistical method that allows for the association of genetic variants with multiple risk factors to be incorporated into the analysis. The approach can be used to adjust for known confounders or to explore the mediating effects of factors that are in the causal pathway from the risk factor of interest to the outcome.
- *MR-Egger* − A common complementary method in MR studies. Can test and adjust for pleiotropy but is sensitive to outliers and less efficient compared to the inverse-variance weighted method.
- *MR-PRESSO (Pleiotropy RESidual Sum and Outlier)* − Can identify and remove outliers but has a high false positive rate with several invalid instrumental variables.
- *Non-linear MR* − A statistical approach to assess the shape of the causal relationship between the exposure and the outcome, and in particular whether the causal effect of the exposure on the outcome varies at different levels of the exposure.
